# Influence of Sociodemographic, Personal, and Lifestyle Factors on Sleep Quality and Stress-Coping Strategies Among Trainees in a Tertiary Hospital

**DOI:** 10.7759/cureus.80294

**Published:** 2025-03-09

**Authors:** Ryan O Alinab, Pearl Angeli B Diamante

**Affiliations:** 1 Department of Neurology, The Medical City, Quezon City, PHL; 2 Department of Neurology, The Medical City, Pasig City, PHL

**Keywords:** coping strategies, lifestyle, physician trainees, shift work, sleep quality

## Abstract

Introduction: Healthcare workers, especially physician trainees, are often required to work demanding shifts exceeding 24 hours, which expose them to various stressors and impact sleep quality and patterns.

Objectives: This study investigated the impact of sociodemographic, personal, and lifestyle factors on sleep quality among hospital trainees and explored their coping mechanisms for managing stress.

Methodology: This analytic, cross-sectional study approved by the hospital's IRB was conducted among hospital trainees (fellows, non-surgical, and surgical residents) in a tertiary hospital. Data were gathered through an online survey using three validated questionnaires: the Pittsburgh Sleep Quality Index, Brief COPE, and FANTASTIC Lifestyle Questionnaire.

Results: Out of 107 respondents, 92 were included in the study, with the majority of respondents being single, female, and having working shifts over 24 hours. The study revealed an 85% (n=79) prevalence of poor sleep quality, with an average sleep duration of 5.7 hours. Sleep duration was significantly associated with working hours and marital status in the non-surgical resident group. Poor sleep quality in general negatively impacted lifestyle factors, especially in surgical residents. Problem-focused coping was the most commonly used strategy, especially among fellows, while surgical residents scored lower in emotion-focused strategies like humor and religion. Avoidant coping, though generally low, was more evident through self-distraction.

Conclusion: This study highlights a high prevalence of sleep deprivation among physician trainees, linked to poor sleep quality and influenced by lifestyle factors. It recommends implementing consistent work shifts, reducing long consecutive hours, and introducing educational programs on sleep hygiene and institutional support to improve trainees' well-being and coping strategies.

## Introduction

Healthcare providers are one of the most demanding service providers in the world. Healthcare organizations usually implement work shifts to cover the intense workload. Some healthcare workers, especially physician trainees, are noted to have work shifts of more than 24 hours. Different work shifts have different exposure to social, physical, and psychological stressors as well as changes in sleep quality and pattern [1]. According to the Joint Consensus of the American Academy of Sleep Medicine and Sleep Research Society, adults should sleep seven or more hours per night regularly to promote optimal health. Sleeping less than the recommended hours is associated with adverse health outcomes, including weight gain and obesity, diabetes, hypertension, heart disease and stroke, depression, and increased risk of death [2]. This study compared the association of sociodemographic (age, gender, marital status, employment status, duration of work shift, body mass index [BMI], comorbidities), personal, and lifestyle factors to sleep quality, patterns, and duration as well as explored the coping mechanisms used to manage stress and improve sleep quality among trainees (surgical resident, non-surgical resident, and fellows).

## Materials and methods

Study design and population

This was an analytic, cross-sectional study done via an online survey form among trainees (residents and fellows) in a tertiary hospital. The study protocol of this research was approved by the hospital’s Institutional Review Board (IRB). Inclusion criteria were full-time or part-time trainees aged 18 years and older, who can read and understand questionnaires written in the English language, trainees on the same shift for the last two months, and employed by the hospital for at least three months. Exclusion criteria include trainees on extended leave of more than two weeks (maternity, sick leaves) as well as those with a history of diagnosed psychiatric disorders (anxiety, depression, bipolar disorders) that can affect sleep, who had less than five shifts in a month, those who exclusively work in the administrative role (without direct patient interaction), and those employed in multiple health facilities concurrently.

Convenient sampling was used to gather the participants and an online self-administered questionnaire was sent to the participants in an online survey form. A letter of request was sent to the Department of Advanced Medical Education (DepAMed). The primary investigators were not able to access the list of trainees of the hospital; hence, the potential participants were directly emailed and messaged by the department to ensure data privacy. The primary investigator communicated with the chief resident/fellow and the senior residents of each department and sent an online survey form, both online and printed QR code, to them to disseminate among the trainees of the department and increase the number of participants. At the beginning of the survey, informed consent was posted, followed by the inclusion and exclusion criteria to screen the participants. Demographic characteristics at the time of the survey, such as age (18-29, 30-39, 40-49, >50), gender (male/female), marital status (single/married, Others), job title (resident, fellow), their specialty (Non-Surgical Specialty [Emergency Medicine, ENT, Family Medicine, Internal Medicine, Neurology, Pathology, Pediatrics, Psychiatry, Radiation Oncology, Radiology]; Surgical Specialty [General Surgery, Neurosurgery, Ophthalmology, Obstetric and Gynecology, Orthopedic Surgery]; and Subspecialty [Cardiology, Colorectal surgery, Endocrinology, Geriatrics, Hematology, Nephrology, Oncology, Pulmonology, Plastics surgery, others]), training, employment status (fully employed/partially employed <6 months), and length of working hours (≤8 hours, >8 hours to ≤12 hours, >12 to ≤24 hours, >24 hours) were collected. Weight, height, and BMI were also included as well as a history of comorbid conditions that were defined but not limited to the following: arterial hypertension (systolic blood pressure or SBP ≥140 mmHg and/or diastolic blood pressure or DBP ≥90 mmHg on repeated examinations or a patient on antihypertensive medication), diabetes mellitus (fasting plasma glucose [FPG] ≥126 mg/dL [7.0 mmol/L], HbA1C ≥6.5% [48 mmol/mol], or a patient on insulin therapy or any oral hypoglycemic medication), dyslipidemia (on maintenance statin therapy or any lipid-lowering medication, or with elevated triglyceride and or low-density lipoprotein [LDL] levels), a previous history of cerebrovascular disease or cardiovascular disease that includes myocardial infarction, valvular heart disease, congestive heart failure, bronchial asthma, chronic obstructive pulmonary disease, and diagnosed sleep disorder such as obstructive sleep apnea.

Sample size

The minimum sample size calculated for the number of subjects was 87 using OpenEpi™ Version 7.1.0.6 Calculator (Centers for Disease Control and Prevention, Atlanta, GA).

Study questionnaires

Validated questionnaires such as the Pittsburgh Sleep Quality Index (PSQI), Brief-Coping Orientation to Problems Experienced Inventory (Brief-COPE), and FANTASTIC Lifestyle Questionnaire (FLS) were used in this study (Appendix). PSQI is a validated 19-item self-report questionnaire with an interval reliability of a =0.83, a test-retest reliability of 0.85 for the global scale, a sensitivity of 89.6%, and a specificity of 86.5%, which assessed various dimensions of sleep quality and disturbances over the past month. The questionnaire has seven component scores such as subjective sleep quality, sleep latency, sleep duration, habitual sleep efficiency, sleep disturbances, use of sleep medications, and daytime dysfunction. Scores for each question range from 0 to 3 and the component scores were summed to obtain a global score ranging from 0 to 21, with higher scores (>5) indicating poorer sleep quality [3]. Brief-COPE Inventory is a 28-item validated questionnaire that assessed effective and ineffective ways to cope, with 14 distinct coping strategies, namely denial, active coping, substance use, behavioral disengagement, use of emotional support, venting, use of instrumental support, religion/spiritual beliefs, positive reframing, planning, self-distraction, humor, acceptance, and self-blame. Each item is rated on a four-point Likert Scale with the first eight scales being adaptive coping strategies while the later six are maladaptive coping strategies. It has good internal consistencies that range from α =0.5 to 0.73 with higher values in factors of religion (α = 0.82) and substance abuse (α = 0.90). Although there was no universally agreed-upon cutoff value defining high scores, a clinical percentile above the median (score of 2) was considered high. The questionnaire was used in various types of individuals such as in healthcare services, and patients with Alzheimer’s disease and cancer [4]. The higher scores indicate greater use of the coping strategy. Currently, there are no questionnaires available that are specific to coping mechanisms for sleep quality. BRIEF-COPE was employed by studies abroad in both healthcare workers and general adult populations that correlated coping mechanisms and sleep [5]. FLS is a validated 28-item questionnaire with nine dimensions, namely F = Family and friends, A = Activity and Associativity, N = Nutrition, T = Tobacco, A = Alcohol and other substances, S = Sleep and stress, T = Type of personality, I = Introspection, and C = Control of health. It has a good internal consistency (Cronbach's alpha = 0.797) with each item question scored from 0 to 2, and a good construct validity (Kaiser-Meyer-Olkin value was 0.786) according to Llorente et al. A total score of 0 to 100 was interpreted as follows: 85-100 as Excellent, 70-84 as Very Good, 55-69 as Good, 35-42 as Fair, and 0-34 as Needs Improvement [6]. After the survey, the investigators provided a brief and informative slide on how to improve sleep hygiene. The primary investigator sent the interpretation via email within 48 hours that included recommendations for further workup and referred to the Employee Health and Wellness Center and Center for Behavioral Health for those assessments with a significant result. Contact details of both services were provided at the end of the survey and provided with the survey result.

Statistical analysis

Descriptive statistics were used to present the distribution of the respondents per sociodemographic profile and sleep pattern between fellows and residents. Test for the significance between the Sleep Quality and Sleep Duration against the Job Title (Fellow, Surgical, and Non-surgical resident) was computed using one-way analysis of variance (ANOVA). Different tests of association were employed for different levels of data of dependent variables in the demographics segment of the questionnaire. Particularly, nominal-level variables with dichotomous responses (1 or 0, yes or no, single or married) are tested using point-biserial and biserial correlation. Point-biserial correlation was used for dichotomous variables “Gender,” “Marital Status,” and “Employment Status.” On the other hand, biserial correlation analyzed the variables “Comorbidity” and “Medications,” dichotomized variables that have other or multiple responses but were analyzed as with/without comorbidity or medication. Additionally, the Kendall Rank Correlation was used for variables with ordinal scales such as “Working Hours” and “BMI.” Finally, the Pearson correlation was used to determine if Sleep Duration and Sleep Quality had a significant association between the FANTASTIC response and its different sub-themes. Pearson correlation was used for testing the relationship between two ratio-level variables.

## Results

Among the 107 respondents who answered the online survey, 15 were excluded from the study. The most frequent reason for dropout was working in multiple healthcare facilities (n=8), followed by not being on a consistent shift for at least two months (n=5) and less than three months employed (n=2). Table 1 showed that most trainees were non-surgical residents (49%, n=45) and from the 30-39 age group (n=56). Most respondents were female (n=59), single (n=74), and fully employed (n=87). A significant portion (60%, n=55) of respondents had worked shifts exceeding 24 hours, while fellows primarily worked within the 8-12-hour range (45%, n=15), though a comparable number (36%, n=12) worked shifts longer than 24 hours. Most trainees (48%, n=44) fell within the normal BMI range, while a substantial proportion (33%, n=30) were classified as overweight. Out of 34 respondents with comorbidities, bronchial asthma (n=14) and dyslipidemia (n=9) were the most prevalent, followed by hypertension (n=4) and diabetes mellitus (n=3). The prevalence of these conditions was generally higher among non-surgical residents. Four reported taking sleep medication (lemborexant, clonazepam, or midazolam) and one participant was using a continuous positive airway pressure (CPAP) machine.

**Table 1:** Demographics: Age, Gender, Marital Status, Employment Status, Length of Working Hours, BMI, Comorbidities

Age	Fellow (%)	Non-Surgical Resident (%)	Surgical Resident (%)	Total (%)
18-29	0 (0)	26 (58)	6 (43)	32 (35)
30-39	30 (91)	18 (40)	8 (57)	56 (61)
40-49	3 (9)	1 (3)	0 (0)	4 (4)
>50	0 (0)	0 (0)	0 (0)	0 (0)
Total	33 (36)	45 (49)	14 (15)	92 (100)
Gender	Fellow	Non-Surgical Resident	Surgical Resident	Total (%)
Female	24 (73)	25 (56)	10 (71)	59 (64)
Male	9 (27)	20 (44)	4 (29)	33 (36)
Total	33 (36)	45 (49)	14 (15)	92 (100)
Marital Status	Fellow	Non-Surgical Resident	Surgical Resident	Total (%)
Single	22 (67)	39 (87)	13 (93)	74 (80)
Married	11 (33)	6 (13)	1 (7)	18 (20)
Total	33 (36)	45 (49)	14 (15)	92 (100)
Employment Status	Fellow	Non-Surgical Resident	Surgical Resident	Total (%)
Fully Employed	28 (85)	45 (100)	14 (100)	87 (95)
Partially Employed	5 (15)	0 (0)	0 (0)	5 (5)
Total	33 (36)	45 (49)	14 (15)	92 (100)
Length of Working Hours	Fellow	Non-Surgical Resident	Surgical Resident	Total (%)
≤8 h	4 (12)	0 (0)	0 (0)	4 (4)
>8 h to ≤12 h	15 (45)	5 (11)	1 (7)	21 (23)
>12 h to ≤24 h	2 (6)	8 (18)	2 (14)	12 (13)
>24 h	12 (36)	32 (71)	11 (79)	55 (60)
Total	33 (36)	45 (49)	14 (15)	92 (100)
Body Mass Index (BMI)	Fellow	Non-Surgical Resident	Surgical Resident	Total (%)
Underweight (<18.5)	1 (3)	1 (2)	0 (0)	2 (2)
Normal (18.5-24.9)	15 (45)	24 (53)	5 (36)	44 (48)
Overweight (25-29.9)	12 (36)	13 (29)	5 (36)	30 (33)
Obese (≥30)	5 (15)	7 (16)	4 (28)	16 (17)
Total	33 (36)	45 (49)	14 (15)	92 (100)
Comorbidities	Fellow	Non-Surgical Resident	Surgical Resident	Total (%)
No Comorbidities	21 (64)	28 (62)	9 (64)	58 (63)
With Comorbidities	12 (46)	17 (38)	5 (36)	34 (37)
Total	33 (36)	45 (49)	14 (15)	92 (100)

Sleep duration, pattern, and quality

The average sleep duration across all participants was 5.7 hours. Non-surgical residents tended to go to bed earlier, with a significant number retiring between 11:00 PM and 12:00 AM, while fellows and surgical residents displayed a more varied bedtime pattern. Non-surgical residents also tended to fall asleep faster, with a large portion drifting off within 15 minutes, whereas surgical residents and fellows exhibited a more varied sleep onset, more often taking more than 30 minutes. Most residents woke up earlier, with a significant number rising between 5:00 AM and 7:00 AM, while surgical residents were more concentrated around 5:00 AM to 6:00 AM.

The majority of respondents (79/92; 85%) reported poor sleep quality, with surgical residents having a slightly higher average PSQI score of 9.14, compared to 8.61 for fellows and 8.47 for non-surgical residents. One-way ANOVA showed no significant difference in the mean hours of sleep duration and quality when grouped by job titles (non-surgical residents, surgical residents, fellows). Table 2 showed that in the conducted tests for association, sleep duration was significantly associated with working hours and marital status among the non-surgical resident group (p-values are significant at a = 0.05). Specifically, non-surgical residents with fewer working hours and who are married tend to have longer sleep durations, and vice versa. Importantly, no significant associations were observed between sleep duration, quality, and other demographic variables when segmented by residents and fellows.

**Table 2:** Test of Association for Sleep Duration and Sleep Quality Against Demographics p-Values are significant at a =0.05.

Sleep Duration	Fellow		Surgical Resident		Non-Surgical Resident	
Comparison Group	Correlation Coefficient	p-Value	Correlation Coefficient	p-Value	Correlation Coefficient	p-Value
Age	0.0877	0.6184	0.1130	0.7190	0.0910	0.5109
Gender	-0.0640	0.7236	-0.3442	0.2281	0.1746	0.2514
Marital Status	-0.0805	0.6558	0.1132	0.6691	0.3304	0.0266
Employment Status	0.0530	0.7697	-	-	-	-
Body Mass Index (BMI)	0.0238	0.8934	0.1290	0.6468	0.0519	0.6991
Working Hours	0.0237	0.8934	-0.2640	0.3430	-0.3200	0.0166
Comorbidity	-0.1776	0.3226	0.0202	0.9451	0.0843	0.5820
Medications	0.1990	0.2669	-	-	-0.1174	0.4424
Sleep Quality	Fellow		Surgical Resident		Non-Surgical Resident	
Comparison Group	Correlation Coefficient	p-Value	Correlation Coefficient	p-Value	Correlation Coefficient	p-Value
Age	-0.0291	0.87423	-0.1460	0.5983	-0.0886	0.4978
Gender	0.0685	0.7051	-0.0960	0.7440	-0.2452	0.1044
Marital Status	0.3984	0.1520	-0.2297	0.4295	-0.2151	0.1559
Employment Status	0.0712	0.6937	-	-	-	-
BMI	-0.1130	0.3667	-0.1400	0.5903	0.0674	0.5923
Working Hours	0.1350	0.3582	0.1140	0.6934	0.1220	0.3380
Comorbidity	0.1560	0.3860	0.3045	0.2897	0.0626	0.6830
Medications	0.0284	0.8754	-	-	0.1472	0.3345

Table 3 presented the analysis of the relationship between sleep duration and respondents' scores on the FLS scores with significance at p-values =0.05, showing no significant correlation between sleep duration and any of the variables or sub-themes. However, sleep quality showed significant correlations with FANTASTIC scores across all groups. Among surgical residents, a moderate negative correlation was found between sleep quality and lifestyle scores. Fellows and non-surgical residents exhibited a weaker negative correlation between these variables, indicating that poor sleep quality is associated with an unhealthy lifestyle, and vice versa. Specifically, fellows showed a weak negative correlation between sleep quality and the "Activity" and "Family and Friends" sub-themes. For non-surgical residents, weak negative correlations were observed between sleep quality and the "Family and Friends," "Nutrition," and "Sleep/Seatbelt/Stress/Safe Sex" sub-themes. Additionally, for surgical residents, a moderate negative correlation was found between sleep quality and the "Insight" sub-theme. These findings suggest that lower sleep quality is linked to poorer lifestyle outcomes across these sub-themes. Notably, a strong positive correlation was observed among surgical residents between sleep duration and the "Family and Friends" sub-theme.

**Table 3:** Test of Association for Sleep Duration and Sleep Quality With FANTASTIC Score p-Values are significant at a =0.05.

Sleep Duration	Fellow		Surgical Resident		Non-Surgical Resident	
Comparison Group	Correlation Coefficient	p-Value	Correlation Coefficient	p-Value	Correlation Coefficient	p-Value
FANTASTIC	0.0146	0.9358	0.4034	0.1527	0.1724	0.2575
Family Friends	0.0998	0.5806	0.7218	0.0036	0.1833	0.2280
Activity	0.0662	0.7143	-0.0810	0.7831	-0.1560	0.3060
Nutrition	0.0652	0.7186	0.2163	0.4577	0.1312	0.3903
Tobacco Toxics	-0.1306	0.4689	0.2191	0.4517	0.0222	0.8846
Alcohol	-0.1653	0.3579	-0.0879	0.7650	0.0910	0.5521
Sleep Seatbelt, Stress, Safe Sex	0.1248	0.4890	0.3538	0.2146	0.2422	0.1090
Type of Behavior	0.0502	0.7814	-0.1158	0.6933	0.1462	0.3378
Insight	0.0678	0.7079	-0.2014	0.4898	0.0243	0.8739
Career	-0.0392	0.8285	-0.0238	0.9355	0.1339	0.3804
Sleep Quality	Fellow		Surgical Resident		Non-Surgical Resident	
Comparison Group	Correlation Coefficient	p-Value	Correlation Coefficient	p-Value	Correlation Coefficient	p-Value
FANTASTIC	-0.3812	0.0286	-0.5707	0.0331	-0.3712	0.0120
Family Friends	-0.3751	0.0315	-0.2759	0.3397	-0.2977	0.0470
Activity	-0.3524	0.0442	-0.1644	0.5745	0.0215	0.8887
Nutrition	-0.2007	0.2627	-0.2070	0.4778	-0.2990	0.0460
Tobacco Toxics	-0.0788	0.6628	0.0086	0.9766	0.0193	0.9000
Alcohol	-0.1610	0.3707	-0.2624	0.3648	-0.2143	0.1575
Sleep, Seatbelt, Stress, Safe Sex	-0.3293	0.0613	-0.4928	0.0734	-0.3992	0.0066
Type of Behavior	-0.0523	0.7723	-0.2884	0.3173	-0.0899	0.5568
Insight	-0.1475	0.4128	-0.5722	0.0325	-0.1672	0.2723
Career	-0.0172	0.9242	-0.3581	0.2087	-0.2708	0.0720

To identify which specific question most significantly correlates with sleep quality, the Pearson correlation coefficient was applied. The main drivers were selected based on two criteria: (1) the question with the highest correlation coefficient and (2) the question with the lowest p-value. For the sub-theme of Nutrition, the main driver for correlation was the question "I often eat excess 1) sugar, 2) salt, 3) animal fats, or 4) junk food." In contrast, within the Sleep/Seatbelt/Stress/Safe Sex sub-theme, "I use seatbelts" was the main driver for significance. Additionally, the question "I relax and enjoy leisure time" also contributed to the sub-theme’s correlation due to its high correlation coefficient and relatively low p-value compared to the leading question (Table 4).

**Table 4:** Correlation Coefficient and p-Values of Specific Questions vs Sleep Quality for Non-Surgical Residents p-Values are significant at a =0.05.

Nutrition		
Specific Question	Correlation Coefficient	p-Value
I eat a balanced diet	-0.2122	0.1617
I often eat excess (1) sugar, (2) salt, (3) animal fats, or (4) junk food	-0.2804	0.0621
I am within _ kg of the weight that I think is healthy	-0.0813	0.5957
Sleep/Seatbelt/Stress/Safe Sex		
Specific Question	Correlation Coefficient	p-Value
I sleep well and feel rested	-0.2797	0.0628
I use seatbelts	-0.3574	0.0159
I am able to cope with the stresses in my life	-0.1746	0.2514
I relax and enjoy leisure time	-0.3127	0.0365
I practice safe sex	-0.1526	0.3170

Coping mechanism

Table 5 presents the average scores (ranging from 1 to 4) from the 92 responses to the BRIEF-Cope questionnaire, which evaluates coping strategies. The findings reveal a preference for adaptive coping strategies across all groups. Problem-focused coping, characterized by proactive efforts to manage stress, was the most common, with fellows achieving the highest mean score (2.78). Emotion-focused coping, aimed at regulating emotional responses, was also widely employed. Avoidant coping, defined by disengagement or avoidance, was the least favored strategy but still utilized to some extent in all groups. When comparing average scores to the overall mean, fellows consistently scored higher across all major coping styles. Both fellows and non-surgical residents showed high scores in problem-focused coping, while surgical residents had relatively lower scores in using informational support and positive reframing. In emotion-focused coping, fellows and non-surgical residents recorded higher average scores across all facets, while surgical residents had lower scores in venting, humor, and religion. Fellows generally outperformed residents in most emotion-focused facets, except for emotional support. Notably, the highest average score (3.09) across all coping styles was for acceptance among fellows. Avoidant coping strategies were relatively low across all groups, except for self-distraction.

**Table 5:** Average score of overarching coping styles across all respondents

	Fellow	Non-Surgical Resident	Surgical Resident	Total
PROBLEM-FOCUSED	2.78	2.52	2.07	2.54
Active Coping	2.95	2.58	2.25	
Use of Informational Support	2.35	2.42	1.89	
Positive Reframing	2.88	2.49	1.96	
Planning	2.94	2.58	2.18	
EMOTION-FOCUSED	2.52	2.37	2.00	2.37
Emotional Support	2.53	2.77	2.32	
Venting	2.24	2.00	1.68	
Humor	2.41	2.28	1.64	
Acceptance	3.09	2.78	2.29	
Religion	2.65	2.27	1.86	
Self-blame	2.20	2.11	2.21	
AVOIDANT	1.79	1.71	1.70	1.74
Self-distraction	2.77	2.96	2.54	
Denial	1.45	1.24	1.25	
Substance Use	1.27	1.10	1.18	
Behavioral Disengagement	1.65	1.54	1.82	

## Discussion

For this study’s summary of results, a survey of 107 trainees, most respondents were non-surgical residents, mainly female, single, and fully employed. About 60% reported working shifts longer than 24 hours, while non-surgical residents generally worked shorter shifts. Poor sleep quality was reported by 85% of participants, with surgical residents experiencing slightly worse sleep than others. For non-surgical residents, sleep duration is influenced by working hours and marital status, though no differences were found between job titles. There was also a weak correlation between sleep quality and certain lifestyle factors, such as nutrition and family relationships. When it came to coping strategies, participants leaned toward healthier, more adaptive methods, especially problem-focused and emotion-focused coping, though fellows tended to use these strategies more effectively than residents. Avoidant coping was less commonly used, and surgical residents scored lower in areas like informational support and positive reframing.

According to the 2018 National Migration Survey, which provided information on the mobility of the Philippine population, less than 1% of all employed working Filipinos are health professionals, a majority of which are nurses (59%), doctors (12%), midwives (11%), pharmacists (6%), dentists (5%), and other health professionals (7%) [7]. Most health professionals have work shifts that can either be morning or night and some even 24 hours or longer, which was observed with the hospital trainees who participated in the study. Healthy sleep depends on the duration, quality, timing, and regularity. Worldwide, the prevalence of sleep deprivation ranges from 1.6% to 56% [8]. The recommended sleep of 7 h or more was not achieved by almost all participants having just an average sleep of 5.7 h. The failure to meet the recommended sleep duration aligns with the findings of the National Health Interview Survey (NHIS), which showed that sleep deprivation among physicians ranges from 29% to 31% in different subspecialties [9]. Similarly, a survey by Wu et al. (2021) found a high prevalence of short sleep duration among physicians working longer hours in China, consistent with our findings [10]. Additionally, a study by Manmee et. al. (2017) also demonstrated a high prevalence of poor sleep quality among trainees, aligning with the results of this study’s result (85%) and the broader context of sleep deprivation among the trainees [11]. Interestingly, there was an association between sleep duration and marital status among the non-surgical resident group. According to Guo et al. (2023), married couples living together have better sleep outcomes compared to unmarried individuals and couples living apart probably due to emotional support and stability [12].

The study suggests that work shifts, particularly night shifts, cause desynchronization of circadian rhythms affecting sleep and contribute to work-related stress, which in turn affects sleep. Residents, particularly in surgery, and fellows have many duties including outpatient department consultations, in-patient rounds, and emergency calls with diverse work shift schedules that often exceed more than 24 hours which may lead to irregular sleep patterns and difficulty in sleep initiation. In this study, fellows and surgical residents tend to have a more spread-out pattern, reflecting their high workload, which suggests a cause-and-effect relationship between workload and sleep quality. This is consistent with the findings from Chang and Li (2022), which noted that night shifts and high work demands negatively impact sleep and stress levels [13].

Physicians generally experience poor sleep quality and shorter sleep durations, which can contribute to unhealthy lifestyle practices such as increased vices, reduced physical activity, unhealthy dietary patterns, and excessive phone or TV screen use [14-16]. This study highlights how these factors contribute to trainees’ overall well-being and stress management. A study by Esen et al. (2017) similarly found that surgical residents experience lower sleep quality compared to other specialties, which impacts their overall lifestyle and cognitive performance [17]. Several studies, including Alghamdi et al. (2024), demonstrated that physicians engaging in physical activity less than once per week had poorer sleep quality [18], while Stanford et al. (2012) found that fellows had lower levels of physical activity compared to residents and medical students, which correlated with poorer sleep quality in this study [19]. This suggests a direct cause-and-effect relationship between physical inactivity, poor coping, and reduced sleep quality. The study also points to the negative effects of poor nutrition on sleep quality. Excessive consumption of processed foods and a lack of fruits and vegetables, as noted by Mota et al. (2013), were linked to poor sleep outcomes among trainees [20]. Poor dietary habits may exacerbate stress and hinder coping abilities, which could create a cycle of poor sleep and increased stress. This underscores the importance of improving nutrition as a preventive measure to enhance sleep quality and coping strategies. Currently, there is no data on seatbelt compliance among physicians; however, a meta-analysis by Kargar et al. (2023) showed that overall compliance was lower, especially among rear-seat passengers than front-seat passengers and those riding public transportation (taxis, buses) [21]. Additionally, studies by Murty and Nayak (2014) revealed that non-surgical residents faced higher work demands and poorer sleep quality, significantly impacting their overall well-being [22]. Jaradat et al. (2022) noted that residents with poor sleep quality reported lower life satisfaction and less leisure time compared to peers in less demanding specialties [23]. Greater social support from family and friends is associated with improved sleep quality and duration among medical trainees, consistent with the findings from a study by Xiao et al. (2020) [24]. Furthermore, the correlation with the "insights" subtheme, which relates to self-awareness about lifestyle choices, suggests that increased stress from this awareness may negatively impact sleep quality among the surgical resident group.

Coping strategies are behaviors that individuals engage in when faced with a stressful situation, to reduce the physical and mental threat that can be expressed as a focus on problem-solving strategies and emotional regulation [25]. The study highlights that hospital trainees employ different coping strategies, with varying levels of effectiveness across groups. Marzo et al. (2021) noted that most Filipino healthcare workers practice positive thinking, high family support, and praying which is consistent with this study [26]. The study reports that hospital trainees exhibited high average scores (2.54) for problem-focused facets, particularly in active coping; however, surgical residents showed relatively lower scores in using informational support (1.89) and positive reframing (1.96) as well as the emotion-focused facets such as venting (1.68), humor (1.64), and religion (1.86). This contrasts with the study of Riaz et al. (2021) which identified planning, positive reframing, acceptance, and religion as the top adaptive strategies and humor, venting, self-blame, and denial as common maladaptive coping strategies [27]. This discrepancy may arise from the surgical training environment where institutional culture may discourage open discussions and seeking support from peers or supervisors, potentially leading to feelings of isolation and frustration [28,29]. This suggests a potential cause-and-effect relationship, where poor coping strategies may contribute to poorer sleep quality, especially among surgical residents. In contrast, non-surgical residents tended to engage in more adaptive coping strategies, which may contribute to better sleep quality.

Cultural dynamics, particularly the Filipino emphasis on resilience and family support, can also influence coping strategies. Litam and Chan (2022) highlighted that while these values can provide emotional support, they may also lead to avoidant coping strategies, such as self-distraction through social media browsing, watching television, or playing video games. This type of coping may serve as a buffer against stress but may also negatively affect sleep quality, as evidenced by the findings in this study [30]. Thus, coping strategies, particularly avoidant coping, can create a negative feedback loop, where inadequate stress management leads to poorer sleep, further exacerbating stress.

## Conclusions

This study exhibited a high prevalence of sleep deprivation and poor sleep quality among physician trainees, with work hours, particularly long shifts, identified as a key factor contributing to sleep disruption. The study suggests a causal relationship between extended work hours, poor sleep quality, and the use of maladaptive coping strategies, especially among surgical residents. The findings show that fellows engaged in more adaptive coping mechanisms, leading to better sleep quality, whereas surgical residents demonstrated lower scores in coping areas like informational support and positive reframing, potentially exacerbating sleep problems. Lifestyle factors including physical activity, nutrition, social support, and life satisfaction, negatively correlated with sleep quality, varied among the subspecialty training programs. Institutional and training culture likely influenced the adaptive and maladaptive coping strategies among physician trainees. The study recommends establishing consistent work shifts and reducing long consecutive work shifts to enhance overall sleep quality. Additionally, educational initiatives on sleep hygiene and improving institutional support systems may foster overall well-being and better coping strategies among physician trainees.

## Figures and Tables

**Figure 1:**
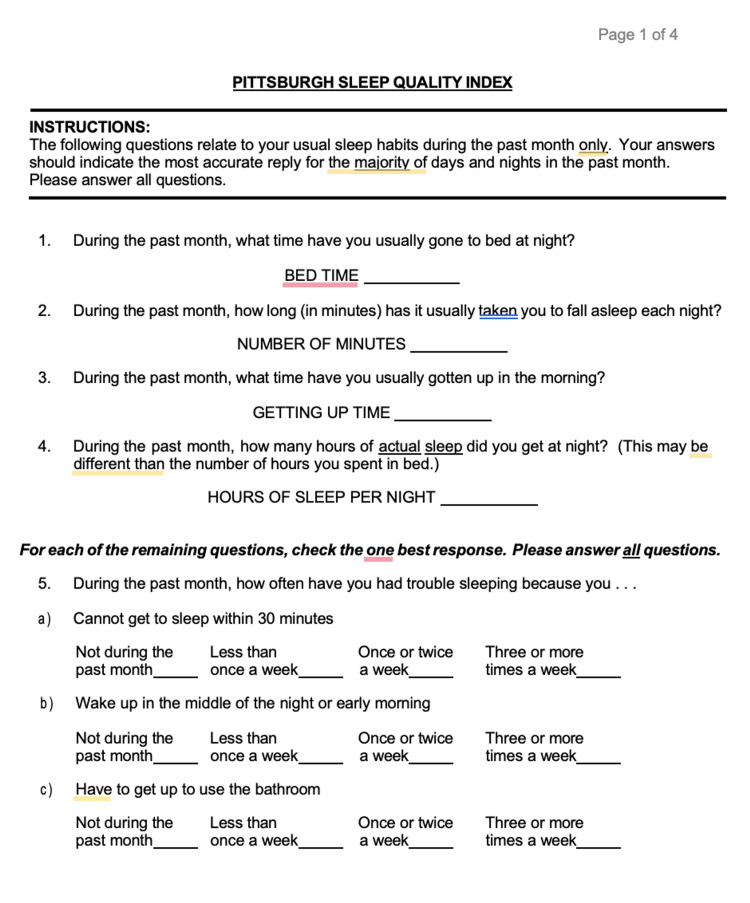
Pittsburg Sleep Questionnaire Index (PSQI) Page 1

**Figure 2:**
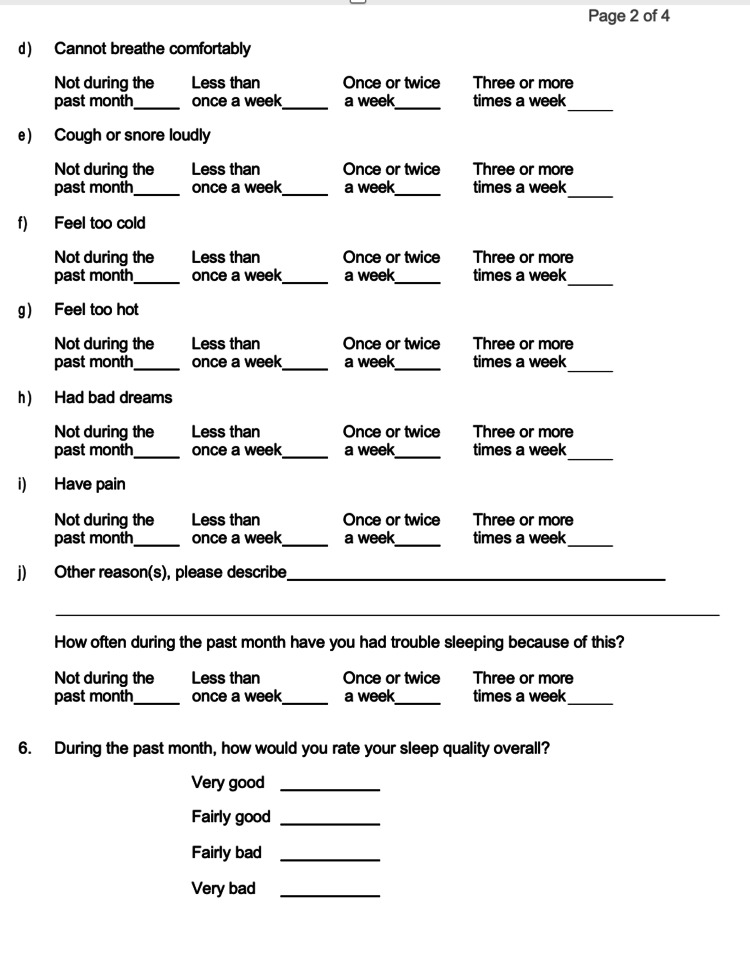
Pittsburgh Sleep Quality Index (PSQI) Page 2

**Figure 3:**
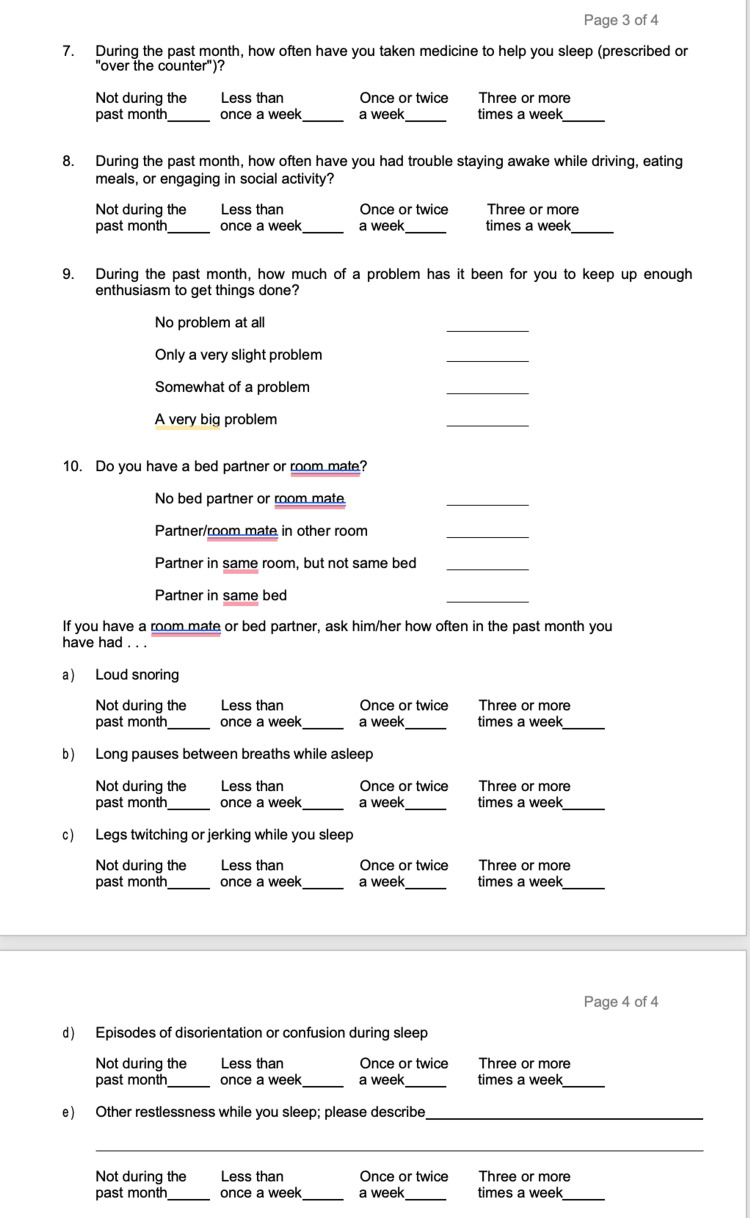
Pittsburgh Sleep Quality Index (PSQI) Page 3

**Figure 4:**
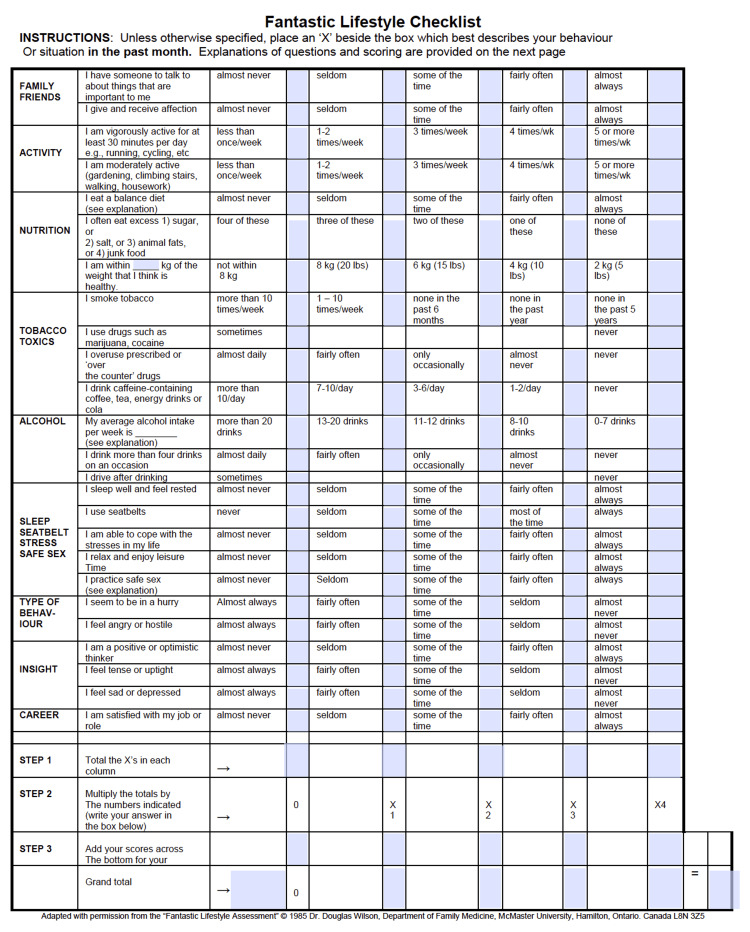
FANTASTIC Lifestyle Questionnaire (FLS)

**Figure 5:**
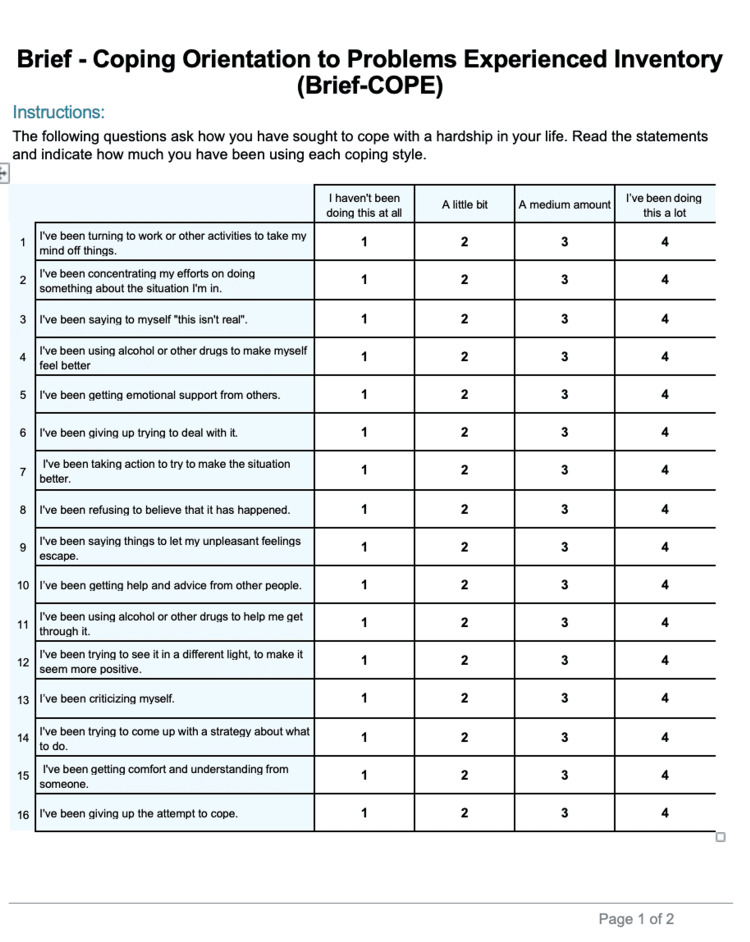
Brief-Coping Orientation to Problems Experienced Inventory (Brief-COPE) Page 1

**Figure 6:**
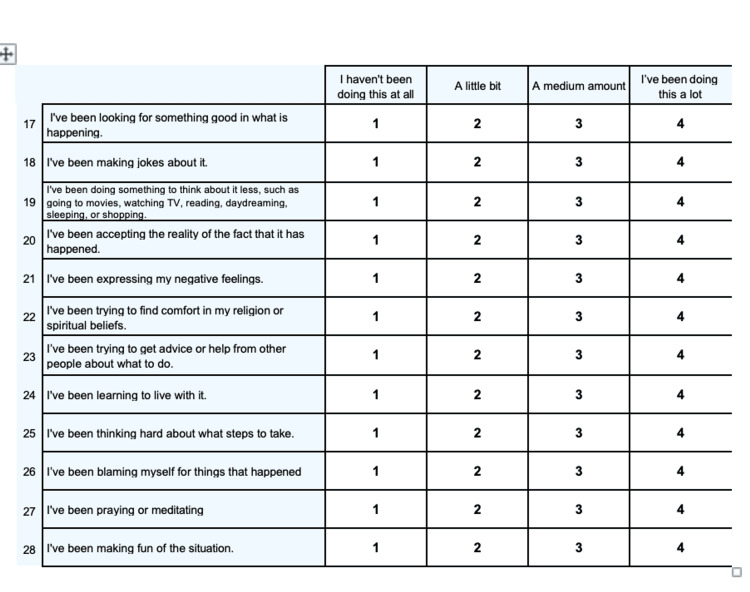
Brief-Coping Orientation to Problems Experienced Inventory (Brief-COPE) Page 2
